# In Memoriam—Mrs. Stephen Seward

**Published:** 1888-09

**Authors:** Frederick Hooker


### IN MEMORIAM.

### MRS. STEPHEN SEWARD.

### MEMORIAL RESOLUTIONS.

At a meeting of the Hahnemannian Club held last night, Drs. William A. Hawley, J. W. Sheldon, and E. J. Robinson, a committee appointed to prepare memorial resolutions on the death of Mrs. Stephen Seward, reported as follows:

WHEREAS, Death has suddenly entered the home of our valued associate and respected President, Dr. Stephen Seward, and taken away his faithful and much loved wife; therefore

*Resolved*, That we extend to him and his family our cordial sympathy in this greatest earthly loss, and that in evidence of this sympathy we will together attend the funeral of Mrs. Seward. Be it further

*Resolved*, That this expression of our sympathy and regard shall be entered on our minutes, a copy thereof sent to the family and published in the daily papers and in THE HOMŒOPATHIC PHYSICIAN.

The resolutions were adopted.

The funeral was held on the afternoon of July 14th, and nearly all the active members of the Club were present.

FREDERICK HOOKER.

